# Detecting Presence of PTSD Using Sentiment Analysis From Text Data

**DOI:** 10.3389/fpsyt.2021.811392

**Published:** 2022-02-01

**Authors:** Jeff Sawalha, Muhammad Yousefnezhad, Zehra Shah, Matthew R. G. Brown, Andrew J. Greenshaw, Russell Greiner

**Affiliations:** ^1^Department of Psychiatry, University of Alberta, Edmonton, AB, Canada; ^2^Department of Computer Science, University of Alberta, Edmonton, AB, Canada; ^3^Department of Computing Science, Alberta Machine Intelligence Institute, University of Alberta, Edmonton, AB, Canada

**Keywords:** post-traumatic stress disorder (PTSD), machine learning, language, emotion, natural language processing, sentiment analysis (SA), telepsychiatry

## Abstract

Rates of Post-traumatic stress disorder (PTSD) have risen significantly due to the COVID-19 pandemic. Telehealth has emerged as a means to monitor symptoms for such disorders. This is partly due to isolation or inaccessibility of therapeutic intervention caused from the pandemic. Additional screening tools may be needed to augment identification and diagnosis of PTSD through a virtual medium. Sentiment analysis refers to the use of natural language processing (NLP) to extract emotional content from text information. In our study, we train a machine learning (ML) model on text data, which is part of the Audio/Visual Emotion Challenge and Workshop (AVEC-19) corpus, to identify individuals with PTSD using sentiment analysis from semi-structured interviews. Our sample size included 188 individuals without PTSD, and 87 with PTSD. The interview was conducted by an artificial character (Ellie) over a video-conference call. Our model was able to achieve a balanced accuracy of 80.4% on a held out dataset used from the AVEC-19 challenge. Additionally, we implemented various partitioning techniques to determine if our model was generalizable enough. This shows that learned models can use sentiment analysis of speech to identify the presence of PTSD, even through a virtual medium. This can serve as an important, accessible and inexpensive tool to detect mental health abnormalities during the COVID-19 pandemic.

## 1. Introduction

Post-traumatic stress disorder (PTSD) is a debilitating condition initiated by exposure to traumatic events, whether witnessing the event in-person, indirectly learning that a traumatic event occurred to a loved one, or through repeated exposure to aversive details of said events (1). There is strong evidence that the current severe acute respiratory syndrome coronavirus two (SARS-CoV-2) pandemic can be considered a global traumatic event (2). Two outcomes have emerged from the SARS-CoV-2 pandemic: (1) There has been a surge in stress-related mental illnesses such as PTSD, specifically in occupational settings (3–7), and (2) many in-person medical appointments have been moved to a digital format (8). Although only a small percentage of individuals develop PTSD following a traumatic event (9), the current pandemic has exposed distressful situations among many. Prior to the global pandemic, a general population survey across 24 countries estimated that 70% of individuals would experience at least one potentially traumatic event (PTE) in their lifetime (1). That figure is now estimated to be higher due to the SARS-CoV-2 outbreak (10). Approximately three in every ten survivors of the SARS-CoV-2 virus, two in every ten healthcare workers, and one in every ten individuals of the general population have reported an official diagnosis of PTSD or PTSD-like symptoms (10). One study estimated that roughly 25% of the general population in the United States has suffered from PTSD during the pandemic (11). With PTSD prevalence rising, it is imperative that we improve on screening and diagnosis, especially with the current emergence of telepsychiatry.

Machine learning (ML) provides a computational tool to better understand the emotional and behavioral nature of PTSD, by learning general rules and patterns from large amounts of patient data (12). With the increasing rate of online assessments, automated identification of disorders such as PTSD can be a useful tool of e-health. Much work has focused on learning a diagnostic classification model that can answer: “Does patient X have PTSD?” Here, a learning algorithm uses a set of labeled instances, to produce a model that uses information about a novel patient (perhaps blood factors, functional neuroimaging, speech, and text data) to predict a “label” value (perhaps “Yes” or “No”) (13). Once trained, a model can make predictions about a novel instance, which we hope are accurate.

Currently, diagnosis of PTSD is done through a clinical interview, which can be inaccurate due to subjective assessments and expertise bias. For example, PTSD is often under-diagnosed and conflated with more prominent disorders such as depression (14), which can affect prognostic outlooks. Second, the etiology of PTSD is multi-causal and complex. Due to the multi-faceted nature of trauma and its kaleidoscopic impact on individuals, clinicians are left to sift through heterogeneous phenotypic expressions (15). This is problematic as: (1) Heterogeneous phenotypic expressions make it difficult for clinicians to assess and treat symptoms of PTSD and (2) differentiating between PTSD and other conditions may be difficult. Next, individuals may feign a PTSD diagnosis for several reasons including legal, personal, social, or financial issues (16). Finally, clinicians also face adversity when calling into question the validity of self-reported trauma or related symptoms, as they may worry about stigmatizing patients or losing rapport with potential victims of trauma (17). Despite the complications, accurate diagnosis can provide patients with adequate treatment earlier, and it can also allow for healthcare systems to properly allocate their resources to those who need it most.

Natural language processing (NLP) is a branch of artificial intelligence that handles text data to decipher and understand human language and context (18). As discussed in Section 2, machine learned models have been applied to text data to accurately identify and treat individuals with, or at risk of, developing PTSD. Natural language has several advantages over other types of modalities such as brain imaging, metabolomics, or genomics: It can be collected at low cost, requiring no more than an audio call, it directly expresses emotions and thoughts through content, it is non-invasive, and it is difficult to conceal and feign symptoms (19). Linguistic content may reveal significant information about an individuals' internal state. Speech is a complex form of communication that interweaves expressive thought, emotion, and intention. It is a window into the mind, and can serve to detect markers of psychiatric illnesses (19–22). Our study uses sentiment analysis (a sub-branch of NLP) to gauge the emotional valence of textual data from an individual suffering from PTSD. Our task is to predict whether an individual may be suffering from PTSD using the emotional valence of their text data in a conversational interview.

In this paper, we use sentiment analysis techniques to detect the presence of PTSD, using text data from a popular dataset, the Audio/Visual Emotion Challenge and Workshop (AVEC 2019) (23), which is a subset of the larger Distress Analysis Interview Corpus of Human and Computer interviews (DAIC_WoZ) (24). The DAIC_WoZ is a multi-modal dataset containing recordings (audio and visual) and transcripts from semi-structured clinical interviews with individuals suffering from PTSD and/or major depressive disorder (MDD), as well as age-and sex-matched controls (24). The protocol was designed to identify people with such disorders. Interviews are conducted by a virtual agent (Ellie) presented on a television screen. Ellie is controlled by a human operator to ask a series of questions to the participants. Two sets of psychiatric questionnaires were used to assess levels of MDD and PTSD (25): the PTSD Checklist-Civilian version (PCL-C) and the Patient Health Questionnaire-Depression 9 (PHQ-9). Our performance task is to predict which individuals are suffering from PTSD using the sentiment/emotional valence from the transcripts provided. The result of the PCL-C questionnaire serves as our outcome variable in this study. This dataset has been examined by previous researchers, who used ML tools to better predict which individuals had MDD. To our knowledge, we have not found any studies which only use text data to predict PTSD in these individuals. Rather, previous studies such as DeVault et al. (26) and Stratou et al. (27) incorporated audio, motion tracking, and text data to predict PTSD. We want to illustrate that emotional language alone can be used to predict PTSD in these individuals, something which has not been done on this popular dataset. However, like those other studies, we plan on incorporating audio and motion tracking data afterwards in a separate study. Thus, the goal of this study is to illustrate that our sentiment analysis can provide accurate predictions, while only use text data on the AVEC-19 dataset, something which has not been accomplished before. We believe this is an important endeavor with the ongoing pandemic and the mental health epidemic happening right now. We also propose that our simple set of features can be used in conjugation with other types of data to improve upon diagnostic accuracy. This may be part of future studies.

Section 2 covers related works using NLP approaches to predict the presence of PTSD in individuals. Section 3 then describes sample demographics and walks through our methods, which include the sentiment analysis pipeline, feature engineering, and the learning procedure. Section 4 discusses the results of our analysis. Lastly, we discuss the space for linguistic analysis in PTSD, and how language can serve as a primary indicator for measuring symptoms.

## 2. Related Work

In recent years, there has been growing interest in building automated systems that could screen for PTSD in individuals. Some approaches involve learned models that use text mining or NLP approaches. A text-based screening tool involves multiple components including data acquisition (an audio recording of an individual's responses to a set of designed questions), feature extraction (quantifying features generated from the dialogue, such as sentiment analysis, bag-of-words, or word embeddings), and classification, which applies a trained ML model to those verbal features in order to predict whether a new patient is suffering from PTSD or not (28). He et al. (29) used lexical features in self-narratives from 300 online testimonies of individuals with PTSD and a control group. Their ML pipeline represented their verbal features using “bag-of-words,” which counts the number of occurrence of keywords in a given document. After, they trained a chi-square model (30) (a method for document classification based on using the chi-square test to identify characteristic vocabulary of document classes) and achieved an accuracy of 82%. He et al. (31) conducted another study examining self-referential narratives about traumatic experiences in a clinical screening process. In that study, they used “N-gram” features (which count the number of co-occurring words within a given window of words). Their Product Score Model with a uni-gram feature space attained an accuracy of 82% over a corpus with 300 individuals (150 with PTSD) who filled out an online survey related to their mental health; this is the highest of all algorithms they tested.

Some feature engineering methods examine the emotion or sentiment of textual data, to produce features that can be used as indicators of several symptoms (32). Language through media such as social media can convey feelings of negativity toward one's self. Large datasets can be derived from social media platforms, which can be useful in training models that can generalize to a wide range of individuals suffering from disorders such as MDD or PTSD. De Choudhury et al. (33) learned a probabilistic model that could detect depression based on Twitter data. The authors generated features based on the emotional sentiment of those tweets, then used dimensionality reduction methods [Principal Component Analysis (PCA)]. Using a support Vector Machine (SVM), they achieved a 74% accuracy in detecting depression. Another study used a pre-trained language analysis tool called Language Inquiry Word Count (LIWC) to extract the emotional polarity of sentences into an overall score. Sentences with the words “mad,” “sad,” “fail,” and “cry” returned a more negative compound score, while words such as “happy,” “joy,” and “smile” returned a positive compound score (34). Though this was not an ML study, they did find significant differences in the linguistic style of individuals suffering from emotional distress compared to those who were not. Another language analysis tool, VADER (Valence Aware Dictionary for sEntiment Reasoning), is a rule-based model that uses both qualitative and quantitative methods to determine the sentiment intensity when humans are verbalizing (35). VADER improves on LIWC by containing a larger word corpus and by being less computationally expensive and more easily implemented. Leiva and Freire (32) used VADER to predict whether someone is at risk of developing depression using sequential social media messages. They reported that VADER was the best sentiment analysis method for predicting whether a user is at risk of depression or not based on the Early Risk Detection Score (ERDS) (32). Sentiment analysis is a useful and simple method to implement on a corpus of text data. Yet, there has been little research done on predicting PTSD using sentiment analysis on an individual basis.

We apply sentiment analysis algorithms such as VADER to the AVEC-19 dataset to determine whether emotional intensity of interview transcripts can serve as predictors of PTSD or not.

## 3. Methods and Analysis

**Figure 1:**
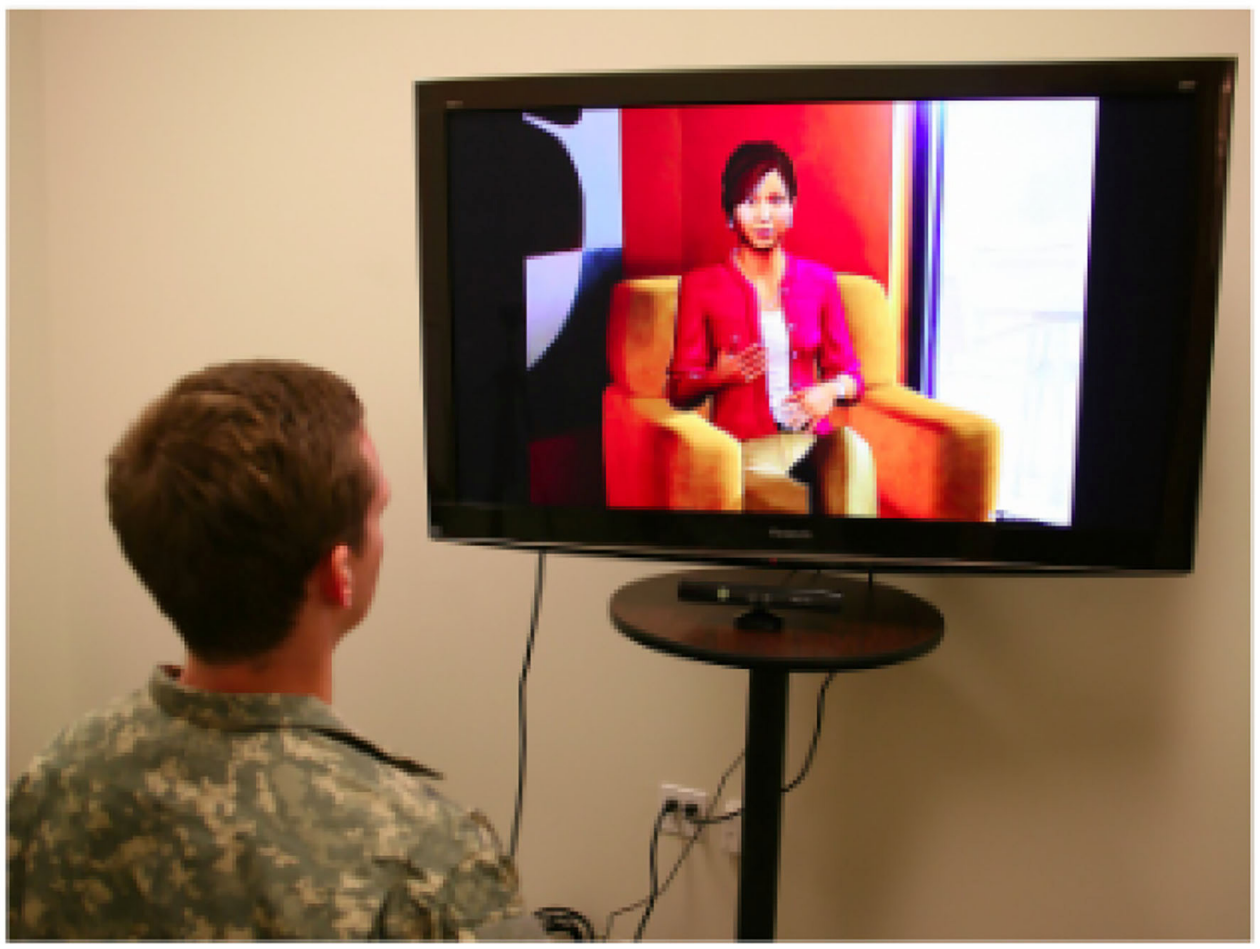
Interview process with Ellie. Participants were placed in a room in front of a large computer screen, showing the animated character Ellie in the Wizard-of-Oz interview.

The DAIC is a large, multi-modal database of semi-structured interviews (24, 36). The original project was started at the University of Southern California (USC), and was approved by the USC Review board (UP-11-00342). The current study includes a secondary analysis of the DAIC dataset, which was designed and collected by Gratch et al. (24) at USC. Our study, which was approved by the University of Alberta's Health Research Ethics Board (Pro 00072946), is a secondary analysis, that did not involve collecting the data nor designing the study. We provide an extensive account of their documented methods below. Individuals with PTSD or MDD participated in a virtual clinical interview with an artificial avatar named Ellie (24). This study was done to compare the development of computer-assisted rates of diagnosis with human performance (24). Ellie was controlled by a human, who administered a series of questions to the individual in a semi-structured manner, while responses were recorded and transcribed to text. Figure 1 reveals the set-up, showing the automated interview with a participant and Ellie. In addition to text, their audio sample was collected, as well as their motion and eye tracking. These interviews were part of a larger project called *SimSensei*, which is developing virtual agents that interview individuals with mental health problems. They are using verbal and non-verbal indicators to screen for cognitive or behavioral abnormalities related to several illnesses (25, 37). Our study only examined the transcribed text data, as our primary focus was to examine whether text data alone could detect the presence of PTSD.

### 3.1. Participants

The DAIC_WoZ dataset includes participants from two populations: U.S. armed forces military veterans (recruited from the U.S. Veterans Facility in Long Beach, New York) and civilians (24, 36), recruited from Craigslist (24, 25). In the DAIC_WoZ subset, participants were flown into the USC Institute for Creative Technologies to participate on-site, in front of a TV. Target participants between the ages of 18-65, who previously had a diagnosis of MDD or PTSD. All participants were fluent English speakers, and all interviews were conducted in English (24). The sample included 275 individuals (105 females, 170 males), 188 controls and 87 who met the criteria for PTSD. Some of those within the PTSD group also met the criteria for MDD. The PTSD Checklist-Civilian version (PCL-C) and the PHQ-9 were used as our outcome metrics (25). We conducted a chi-square test to determine whether if the sex ratio was significantly different between the two groups. We also conducted a two-sample *t*-test for testing the mean PCL-C and PHQ-9 scores between the two groups. Note that the PCL-C is not used for official diagnosis of PTSD, but it is strongly correlated with the Clinician Administered PTSD Scale (CAPS-5), which is the gold standard measurement for diagnostic efficacy (38). Table 1 illustrates the symptoms scores for both of these questionnaires across the control and target groups.

**Table 1:** Demographics and outcome measures.

	**Non-PTSD**	**PTSD**	**Test score value**	**Significance**	
Sex (Male / Female)	122 / 66	48 / 39	*X*_(1, 1)_= 2.38	N/S	
PTSD mean score (PCL-C)	26.54 (± 8.77)	57.98 (± 10.70)	*t*_(273)_= 24.06	*p* < 0.0001	
Depression mean score (PHQ-8)	4.177 (± 3.65)	15.69 (± 3.48)	*t*_(273)_= 23.13	*p* < 0.0001	

*Clinical and demographic descriptions of both groups (Non-PTSD and PTSD individuals). For testing sex differences across both groups, a chi-squared test was used. A t-test was conducted on the group means and standard deviations of both the PCL-C and the PHQ-8 scores to determine if they were statistically significant. Standard deviation can be seen within the brackets of both assessment tests. Statistical significance was set at p <0.05. N/S refers to not statistically significant*.

### 3.2. Procedure

Prior to the recorded interview, participants were given an explanation of the study, and then voluntarily signed a consent form (24). Then, a series of questionnaires were conducted online, which included a demographics section, the PCL-C and the PHQ-9.

After completing the questionnaires, participants sat in front of a virtual character (Ellie), who was projected on a 50-inch T.V. monitor (24) as seen in Figure 1. Participants were recorded on a Logitech 720p webcam, and used a Sennheiser HSP 4-EW-3 microphone (24, 25), audio recording at 16 kHz. Acoustic data was recorded and stored by SimSensei (37). For text data collection, SimSensei used the PocketSphinx recognizer to recognize spoken words for Ellie and the participants, and saved them in a document (25, 37, 39). The individuals controlling Ellie used the Flores Dialogue Manager to decide on the proper responses and questions to ask the participants (40). Ellie first explained the purpose of the study, then asked a series of “ice-breaker” questions to build rapport with the participants (24, 25). It then asked a series of emotionally valenced questions, such as: “What are some things that put you in a good mood?” or “What are some things that make you mad?” as well as some neutral questions (25, 36). Ellie also provided supportive responses (i.e., “That's great” or “I'm sorry”) that were used in a balanced manner throughout the interviews (36). The questions and animated movements of Ellie were pre-recorded and designed using SmartBody, a software from USC that automates physical and verbal reactions for virtual humans (25). After the interview was completed, they were then debriefed and given $35 as compensation for participating.

#### 3.2.1. Transcription

**Figure 2:**
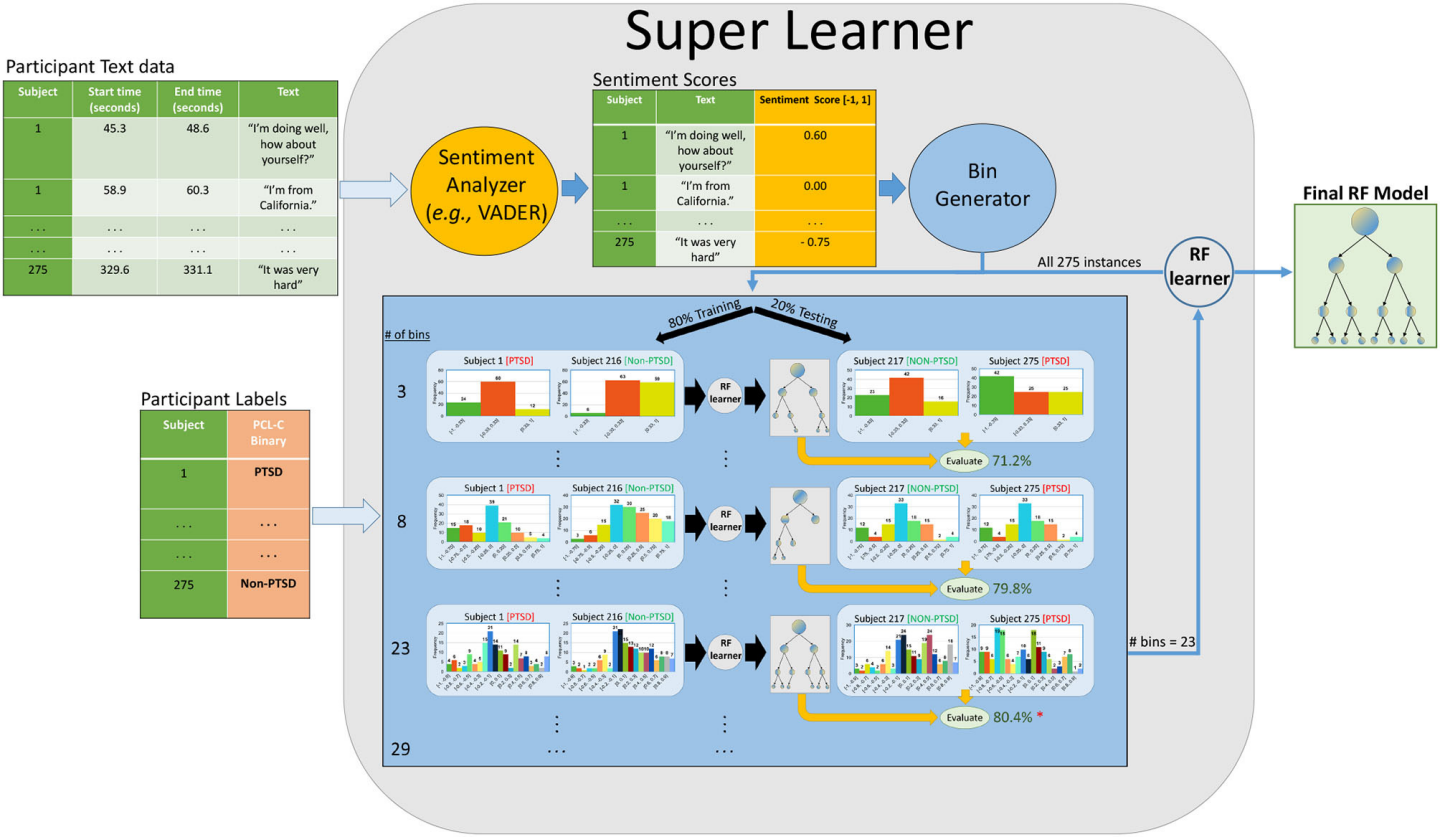
Sentiment analysis pipeline. A simplified version of our pipeline. Raw text is given to a sentiment analyzer (i.e., VADER/Textblob/Flair) that outputs a compound scalar score between [−1, 1] for each utterance. Note that each participant provides many such utterances in the session; our SuperLearner (SL) then bins that participant's set of scores into a set of k bins. It uses internal cross-validation (on the training set) to identify the optimal number of bins and tune hyperparameters. Here, it found that 23 bins was optimal. We repeat this process with different partitioning methods with our dataset, such as five-fold-CV and the original train-test folds. We also consider four different base learners, though our figure only shows the RF learner [Linear Discriminant Analysis (LDA), Support Vector Classifier (SVC), and Random Forests (RF), and Gradient Boosting (GB)].

Transcripts were transcribed and segmented by the ELAN tool from the Max Planck Institute for Psycholinguistics (41). The transcriptions were segmented into utterances based on audio boundaries with at least 300 ms of silence in the recordings. Timestamps display the length of utterances, as seen in Figure 2. In the current DAIC_WoZ dataset, Ellie's responses were removed from the transcribed data (36).

All transcribed interviews underwent de-identification. Human annotators scanned all utterances for mentions of names, dates, and places that could be used to narrow down an event and replaced them with special tokens (24). Both transcriptions and de-identification were also performed by two independent annotators, and transcription discrepancies were handled by a senior annotator.

#### 3.2.2. Preprocessing

Preprocessing for sentiment analysis can differ depending on the type of analyzer being used. We consider both rule-and embedding based analyzers. Some rule-based analyzers, such as VADER, handle most preprocessing steps, with an emphasis on the lexical nature of a given document. Since VADER compares the performance of its parsimonious model against human-centric baselines, the preprocessing pipeline involves limited text cleaning; see Hutto and Gilbert (35) for the VADER preprocessing pipeline. Embedding-based analyzers, such as FLAIR, require text embeddings, which represents each word as an *n*-dimensional vector, where similar words tend to be closer together in this *n*-dimensional space. Such embedding based analyzers usually involve stemming, lemmatizing, removing special characters, generating word tokens, and word embeddings (42). For our study, we performed these preprocessing steps for FLAIR, but allowed rule-based analyzers (VADER and TextBlob) to perform these steps with their own internal functions. Regardless of the analyzer used, we employed basic text cleaning, which involved removing any brackets or quotations, digits, stop words, and any upper case letters. We also removed the first five utterances of every participant's data, as the transcribed text involved conversations between the experimenters and the participant shortly before the interview began.

#### 3.2.3. Sentiment Analysis

For each utterance from a given subject, our system produced a sentiment score based on the emotional polarity and intensity of the utterance (35). Three different sentiment analyzers were considered for this study. VADER is a ruled-based model that uses lexical, grammatical, and syntactic rules for expressing the sentiment polarity and intensity of a text. It relies on a dictionary of words that map lexical features to sentiment scores based on emotionality (35, 43). The VADER lexicon performs better than individual human raters (*F1* = 0.96 vs. *F1* = 0.84) at correctly classifying sentiment of tweets into positive, neutral, or negative classes (ground truth is an aggregated group mean from 20 human raters) (35). For a given utterance, VADER generates a compound score between [-1, 1], indicating the overall sentiment and the intensity. Phrases such as “This is horrible” will have compound scores near -1, while “I am so happy!” will have compound scores near 1. The second sentiment analyzer was FLAIR: An easy-to-use framework for state-of-the-art NLP, a pre-trained language model that uses a mix of supervised and unsupervised techniques to capture sentiment content by examining word vectors (44). FLAIR relies on a vector representation of words to predict sentiment annotations depending on the order of those words. Like VADER, FLAIR outputs a sentiment polarity score between [-1, 1] for a given document (44). Lastly, TextBlob is a rule-based analyzer, that uses a pre-trained set of categorized words. Sentiments are defined based on the semantic relation and frequency of each word in a document (45). TextBlob also outputs a polarity score between [-1, 1].

#### 3.2.4. Machine Learning

We generated a sentiment score for each utterance in our dataset. For each participant, we binned their sentiment scores into evenly distributed ranged bins from [-1, 1]. In predictive modeling, binning is done to transform continuous values into intervals, with the hope of optimizing the stability of predictive performance. It can also reduce statistical noise or complexity in the variables (46, 47). We chose this method because we wanted to normalize the length of transcripts across all participants. By binning sentiment scores, we were able to concatenate the entirety of our features into one row per subject. There is also evidence stating that certain classification models can benefit from binning numeric values (48). For our study, we used unsupervised binning, which places variables into bins of equal range (46). For example, if we wanted four bins of sentiment scores for a given subject, we would place sentiment scores from his/her utterances into these bins: [-1, -0.5, 0, 0.5, 1]. We treated the number of bins as a hyper-parameter in our ML pipeline, and let our learner select the optimal number of bins (# bins ∈[3, 6, 7, 8, 9, 12, 15, 18, 21, 23, 25, 26, 29]) based on our evaluation metrics in the training set. The bin sizes were randomly generated between 2 and 30 to cover a large parameter space for our learner. Bin sizes below 3 and above 30 were not considered because they did not resemble a normal distribution for compound scores. Sentiment binned scores were not normalized based on the number of utterances for this pipeline. Instead, we used bin discretization to normalize the number of features. Thus, we do not account for length of transcripts between groups, as we believe that is a relevant feature for this study. As a result, we use a super learner (SL) that combines base learners [Random Forests (RF), Gradient Boosting (GB), Linear Discriminant Analysis (LDA), SVM], and model configurations on the same split of data, and then uses out-of-fold predictions to select the best configurations or models (49, 50). Our SL selects the optimal bin size (as a sort of hyperparameter), the best performing base learner, along with its hyperparameters, then runs the resulting learner (with the chosen settings), on the training dataset, to produce a final classifier. Figure 2 shows a simplified version this pipeline that involves the RF algorithim.

We devised many partitioning techniques on the same dataset to determine whether our models were generalizable. Originally, the DAIC_WoZ dataset used a pre-determined training, validation, and test set, since it was part of an official data science competition (36). However, for clinical purposes, we opted to use different partitioning techniques with all the data such as: Original folds (Train-test-split) from the AVEC-19 competition, nested cross validation (five-fold-CV), and training on participants from the 2017 edition and testing on 2019 participants (2017-to-2019). Regardless of the partitioning type, our learner used internal five-fold-CV to optimize the parameters of a model on a training fold to produce a model that is then evaluated on a held-out fold (51). We averaged the accuracies of the test folds in our analysis. Using multiple partitioning methods help us understand the nature of the data, and eliminates the chance of a biased predefined train and test set. We wanted to be sure that this model could generalize to unseen data, hence the motivation to implement different partitioning techniques. For each model, we ran all partitioning types and compared accuracies between them.

One central issue that arises is the imbalanced class sizes between individuals with vs. without PTSD. For each fold, regardless of the partitioning type, we randomly over-sampled the minority class in the training set if the majority class had 10% more instances compared to the minority class (52). Here, we chose minority class samples at random, with replacement using the “imbalanced learn” package for Python (Version 0.8.0). This was done to increase the sensitivity of our model, and reduce the number of degenerate predictions from non-PTSD individuals.

Our SL considered three different base learners, and hyper-tuned each one with its various parameter settings. Regardless of how we partitioned the data, we only used the training data to hyper-tune the parameters. First, we used a RF learner. An RF is an ensemble ML method that constructs various decision trees, first training each decision tree on a random “bagged” subset of the data. After learning this RF model, at performance time, the instance is dropped in each of the trees. These RF models have been used extensively in speech analysis for identifying PTSD and depression (53–55). One study used an RF on the AVEC-17 dataset, which only included PHQ-8 scores, and achieved a precision score of 0.68 (recall = 0.65) using only text data (55). Another study looking at depression on Twitter used an RF with VADER, and reached a precision of 0.19, but a recall of 0.96 (32). Lastly, one study used a combination of proprietary sentiment analyzers (labMT, LIWC and ANEW) with an RF on 243,000 tweets (produced by 63 users) of individuals with and without PTSD labels (53). Their RF achieved an AUC of 0.89.

The second base learner is a Support Vector Classifier (SVC), which learns the separating plane that maximizes the distance to the support vectors (56). Traditionally, SVCs have worked well with text data, perhaps due to their non-linear flexibility due to kernels. However, we only use linear kernels due to the nature of our binned data. He et al. (31) used an SVC as one of their models in an n-gram based classification of individuals with PTSD vs. without. The uni-gram feature set with an SVM achieved an accuracy of 80% (31). Leiva and Freire (32) also used an SVC with VADER. After using PCA, their SVC achieved a precision of 0.58, and a recall of 0.56 (32).

Next is GB, which has been used in sentiment analysis for social media text, particularly related to mental health (57–59). Gradient Boosting is a sequential boosting method that uses large trees that concentrate on misclassified observations, found by using the gradients of large residuals computed in previous iterations to refine future predictions (60). A recent paper examined detecting anxiety based on social media data related to the COVID-19 pandemic. Their study used sentiment analysis with a number of different models, including Extreme Gradient Boosting (XGB) (59), which is a stochastic version of GB, and is computationally faster for large datasets. Their XGB model achieved an accuracy of 73.2 %, but had the highest recall with 0.87 against other models such as K-nearest neighbors, SVC, RF, and decision trees (59). Another study looked at detecting anxiety and depression from social media data using word frequencies, timing, and sentiments (58). Though their RF model achieved the best accuracy, the GB model achieved an accuracy of 79.1 %, and the results were combined in an ensemble voting approach, which achieved an accuracy of 85.1%.

Our last base learner is LDA. This method maximizes the ratio of between-class variance to within-class variance in any dataset, in an attempt to maximize separability (61). It has been used as both a dimensionality reduction method for variables and a classification model. Linear Discriminant Analysis makes predictions by estimating the probability that unseen inputs belong to one of two distributions. The model will classify the input based on the highest probability between the two distributions (61).

The hyperparameter space is as follows: (1) SVC: “loss”: [“hinge,” “log,” “squared_hinge,” “modified_huber”], “alpha”: [0.0001, 0.001, 0.01, 0.1, 1, 10, 100], “penalty”: [“L2”, “L1”, “elasticnet”, “none”]. (2) GB: “learning_rate”: [0.0001, 0.001, 0.01, 0.1, 10, 100], “n_estimators”: [50, 100, 500]. (3) RF classifier: “max_depth”: [5,10, 15], “max_features”: [2, 3], “min_samples_leaf”: [2, 3, 4, 7], “min_samples_split”: [8, 10, 12], “n_estimators”: [100, 200, 300, 500]. (4) LDA: “solver”: [“svd”, “lsqr”, “eigen”], “tol”: [0.0001, 0.0002, 0.0003, 0.1, 0.01, 0.5, 0.0009, 0.09]. The classification metric used to evaluate the models were accuracy, but we also report area under curve (AUC), F1-score, recall and precision. We plotted the average score from the external folds in all sentiment analyzers and all models, which can be seen in the results section. To summarize, we compared four different models with three different types of cross-validation, using three different sentiment analyzers.

## 4. Results

### 4.1. Demographics

Table 1 summarizes the clinical and demographic characteristics of both groups. When examining the number of males and females in both groups, the chi-square test returned an insignificant chi-square value [*X*_(1, 1)_ = 2.38, *p* > 0.05]. For the PCL-C and PHQ-8 scores, a *t*-test was used to determined if there was a difference between the non-PTSD and PTSD group means. There was a significance difference for the PCL-C between groups [*t*_(273)_ = 24.06, *p* < 0.0001], and there was a significant difference in the PHQ-8 test [*t*_(273)_= 23.13, *p* < 0.0001].

Table 2 summarizes the clinical and demographic characteristics from the original AVEC-19 training and testing sets. When examining the number of males and females in both sets, the chi-square test returned an significant chi-square value [*X*_(2, 1)_= 10.19, *p* < 0.001]. For the PCL-C scores, a one-way ANOVA was used to determined if there was a difference between the non-PTSD and PTSD group means. There was a significant difference for the PCL-C between groups [*F*_(3, 271)_ = 227.04, *p* < 0.0001].

**Table 2:** Demographics and outcome measures for original partitioning folds.

	**Training**			**Testing**				
	**Non-PTSD (*n* = 153)**	**PTSD (*n* = 66)**		**Non-PTSD (*n* = 35)**	**PTSD (*n* = 21)**		**Test score value**	**Significance**
Sex (Male / Female)	93 / 60	34 / 32		29 / 6	14 / 7		X_(2, 1)_ = 10.19	*p* < 0.05
PTSD mean score (PCL-C)	26.23 (± 8.47)	56.44 (± 10.29)		27.94 (± 10.06)	63.05 (± 10.70)		F_(3, 271)_ = 227.04	*p* < 0.001

*Clinical and demographic descriptions of both groups (Non-PTSD and PTSD individuals) in the original train-test partition from the AVEC-19 challenge. For testing sex differences across training and testing sets, a chi-squared test was used. A t-test was conducted on the group means and standard deviations of PCL-C scores to determine if they were statistically significant. Standard deviation can be seen within the brackets of both assessment tests. Statistical significance was set at p <0.05. N/S refers to not statistically significant*.

Table 3 summarizes the clinical and demographic characteristics from the participants included in the 2017 and 2019 versions. Here, the unique individuals from the 2017 version represented the training set, and individuals only from the 2019 version represented the testing set. When examining the number of males and females in both sets, the chi-square test returned a significant chi-square value [*X*_(2, 1)_ = 19.19, *p* < 0.001]. For the PCL-C scores, a one-way ANOVA was used to determined if there was a difference between the non-PTSD and PTSD group means. There was a significant difference for the PCL-C between groups [*F*_(3, 271)_ = 230.04, *p* < 0.0001].

**Table 3:** Demographics and outcome measures for 2017-to-2019 partitioning folds.

	**Training**			**Testing**				
	**Non-PTSD (*n* = 133)**	**PTSD (*n* = 56)**		**Non-PTSD (*n* = 55)**	**PTSD (*n* = 31)**		**Test score value**	**Significance**
Sex (Male / Female)	77 / 56	25 / 31		45 / 10	23 / 8		X_(2, 1)_ = 19.20	*p* < 0.001
PTSD mean score (PCL-C)	26.42 (± 8.74)	55.82 (± 10.66)		26.83 (± 8.93)	62.00 (± 9.68)		F_(3, 271)_ = 230.04	*p* < 0.001

*Clinical and demographic descriptions of participants from the 2017 (training set) and 2019 competition (testing set) (Non-PTSD and PTSD individuals). For testing sex differences across training and testing sets, a chi-squared test was used. A t-test was conducted on the group means and standard deviations of PCL-C scores to determine if they were statistically significant. Standard deviation can be seen within the brackets of both assessment tests. Statistical significance was set at p <0.05. N/S refers to not statistically significant*.

### 4.2. Machine Learning and Statistical Analysis

**Figure 3:**
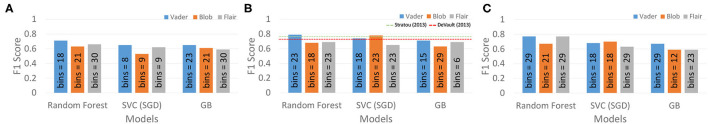
Machine learning results (F1 score) from partitioned types. **(A)** Five-fold-CV, **(B)** Original Train-Test-Split, and **(C)** 2017-to-2019. High watermark results from each model, sentiment analyzer and bin size for all 3 partitioning methods. The RF (23 bins) with VADER on the original train-test-split folds achieved the highest accuracy (80.4 %) and an F1 score (0.79). Panel **(B)** displayed the benchmark F1 scores from two other studies. In the agnostic five-fold-CV, the RF (18 bins) with VADER achieved the best accuracy (75.6 %, STD = ± 4.5 %, F1 score = 0.71). The dashed lines represent the results from Stratou et al. (27) and DeVault et al. (26), who tried to predict PTSD on a smaller version of this current dataset.

Figure 3 reveals the F1 scores of each model and sentiment analyzer between the various partitioning methods. In the five-fold-CV procedure seen in Figure 3A, the RF model with VADER returned a mean accuracy of 75.6 % (± 4.5 % STD). The AUC was 0.72, and the F1-score was 0.83 and 0.58 for the non-PTSD and PTSD groups. The precision was 0.70, and the recall was 0.67. The optimal number of bins for this model was 18 bins based on the accuracy of the training set occurring in the grid search. Generally, the RF model outperformed all other models with all sentiment analyzers; see evaluation metrics in Table 4. Figure 3B shows the results with respect to the traditional train-test-split sets from the AVEC-19 competition. The high watermark from our analysis was found here. The RF using VADER, with 23 bins, revealed the highest mean accuracy (80.4 %), with an AUC of 0.80, and an F1-score of 0.85 and 0.72 for the non-PTSD and PTSD groups. The precision was 0.84 and the recall was 0.75. The RF also achieved a high accuracy with the 2017-to-2019 partitioning split (80.2 %), with both the VADER and flair sentiment analyzers (bins = 23 for both). We found two benchmark studies to compare our results to. One study was from Stratou et al. (27), who achieved an F1-score of 0.79 on their Naive Bayes model, with only 53 participants. However, this model incorporated audio, motioning tracking, and text data together. The second study from DeVault et al. (26) achieved an accuracy of 74.4% (F1 score = 0.738) on the same 53 participants. They also used audio and textual features. The second-highest performing model was the SVC with 23 bins, using Textblob (accuracy = 78.6 %), AUC = 0.78, F1-score: non-PTSD = 0.81, F1-score: PTSD = 0.75, precision = 0.77, recall = 0.75). Figure 3C used participants from the AVEC-2017 cohort to train, and tested on individuals only from 2019. Overall, models using VADER seemed to garner the best results compared to the other analyzers as seen in Table 4 (VADER = 73.2 % mean, Flair = 70.3 %, Textblob = 69.7 %, and the RF seemed to outperform GB and SVC. Lastly, the train-test-split partitioning type had higher accuracies compared to the other partitioning methods (train-test-split mean accuracy = 74.0 %, 5-CV = 69.1 %, 2017-to-2019 = 70.2 %).

**Table 4:** Performance of models across sentiment analyzers and partitioning schemes.

**Model**	**Partition type**	**Sentiment analyzer**	**Bins**	**Accuracy mean ±std**	**AUC**	**F1: Control**	**F1: Target**
Random forest	Train-test-split	**Vader**	**23**	**80.4**	**0.80**	**0.85**	**0.72**
		Flair	23	75.0	0.80	0.82	0.53
		Textblob	12	71.4	0.70	0.79	0.56
	Five-fold-CV	Vader	18	75.6 ± 4.5	0.72	0.83	0.58
		Flair	30	74.0 ± 2.9	0.70	0.82	0.49
		Textblob	21	70.2 ± 6.3	0.65	0.79	0.45
	2017-to-2019	Vader	23	80.2	0.82	0.86	0.67
		Flair	23	80.2	0.81	0.86	0.67
		Textblob	21	72.1	0.71	0.80	0.52
Support Vector Machine (SVM)	Train-test-split	Vader	18	75.0	0.73	0.80	0.67
		Flair	23	70.0	0.68	0.78	0.51
		Textblob	23	78.6	0.78	0.81	0.75
	Five-fold-CV	Vader	8	70.0 ± 4.9	0.66	0.77	0.51
		Flair	9	66.2 ± 2.9	0.62	0.75	0.47
		Textblob	7	67.3 ± 4.6	0.62	0.76	0.46
	2017-to-2019	Vader	18	70.1	0.68	0.77	0.59
		Flair	29	68.6	0.65	0.78	0.47
		Textblob	18	70.0	0.70	0.74	0.65
Linear Discriminant Analysis (LDA)	Train-test-split	Vader	3	73.0	0.77	0.74	0.72
		Flair	18	75.0	0.75	0.82	0.61
		Textblob	29	75.0	0.75	0.78	0.71
	Five-fold-CV	Vader	6	70.2 ± 2.9	0.67	0.77	0.58
		Flair	30	64.3 ± 2.5	0.60	0.73	0.46
		Textblob	9	65.5 ± 6.6	0.61	0.74	0.48
	2017-to-2019	Vader	18	67.4	0.65	0.75	0.55
		Flair	6	63.9	0.62	0.71	0.52
		Textblob	26	67.4	0.65	0.74	0.58
Gradient Boosting (GB)	Train-test-split	Vader	15	75.0	0.74	0.81	0.63
		Flair	6	73.2	0.73	0.81	0.57
		Textblob	29	66.1	0.63	0.74	0.52
	Five-fold-CV	Vader	23	70.5 ± 3.2	0.66	0.79	0.49
		Flair	29	67.3 ± 2.5	0.60	0.77	0.41
		Textblob	21	67.6 ± 6.2	0.62	0.77	0.45
	2017-to-2019	Vader	29	70.9	0.68	0.78	0.56
		Flair	23	66.2	0.62	0.76	0.40
		Textblob	12	65.1	0.61	0.75	0.42

*A list of best performing models based on the highest accuracy across the various bin sizes. The high watermark model was the RF using the VADER analyzer, with 23 bins, and the traditional train-test-split partition. This is denoted by the bolded values in the table*.

### 4.3. Bin Analysis

**Figure 4:**
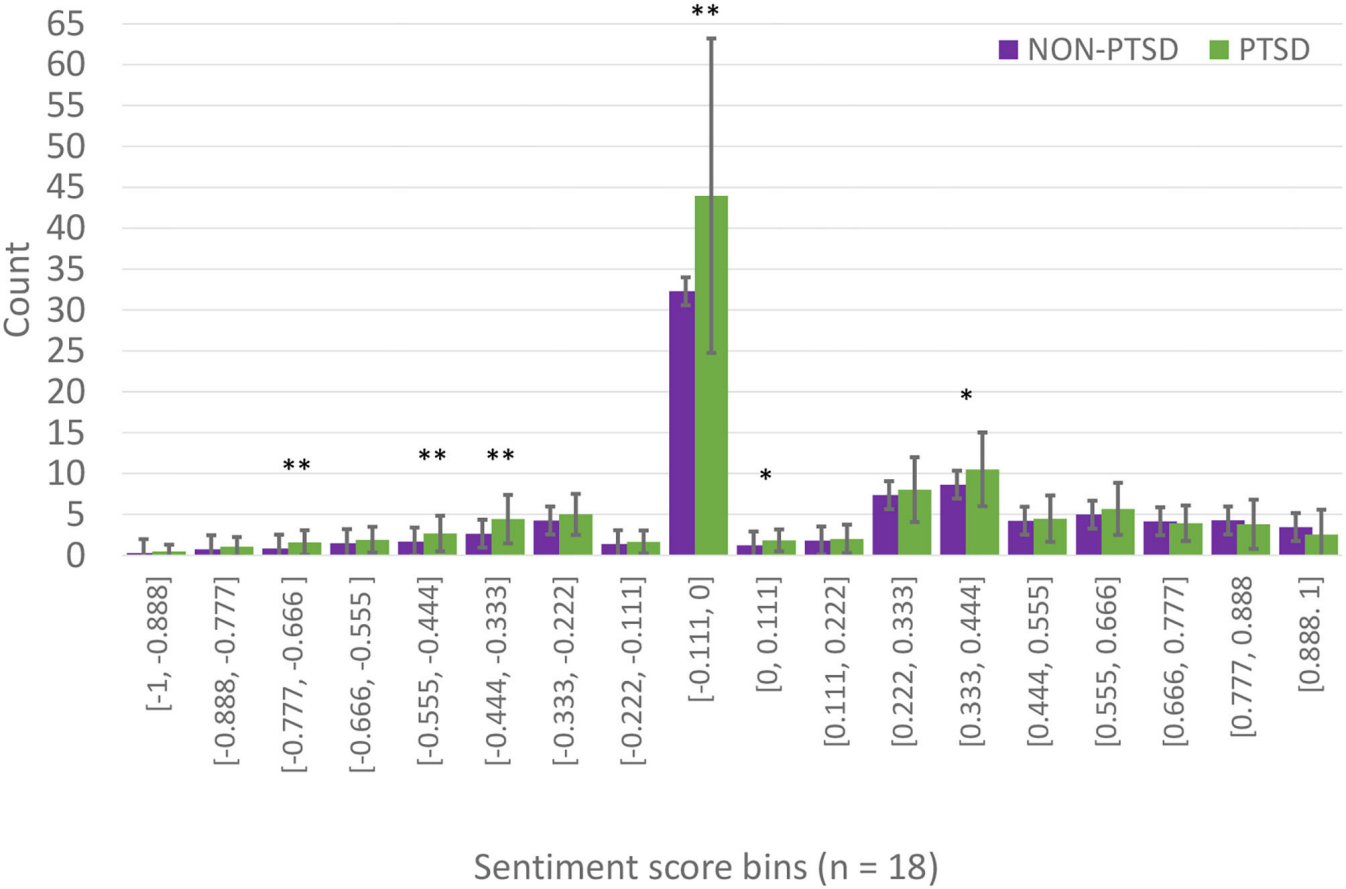
Binned sentiment scores per group. These are the mean values of chosen binned sentiment scores (from the RF model in the five-fold-CV partition), in each bin, for both groups. The x-axis represents the bins and their sizes, and the y-axis represents the average number of utterances that fall within those sentiment scores for both PTSD and non-PTSD groups. Table 5 shows the means and standard deviations, and a one-way ANOVA was conducted to compare these values in each bin between the two groups. A Benjamini-Hochberg correction was made for 18 comparisons. The adjusted *p*-value threshold was set to *p* < 0.00284. Error bars represent standard deviation. Significant difference denoted by *p < 0.00284, **p < 0.0001.

Next, we looked at the number of sentiments across all 18 bins (the winning chosen bin size from the 5-CV partitioning method) from our SL. In Figure 4 and Table 5, the number of utterances within a certain interval of sentiment scores was plotted across both groups. For the PTSD group, there was a higher number of negative sentiments compared to the Non-PTSD group. As you move toward positive sentiments, the Non-PTSD group contained more extreme positive utterances compared to the PTSD group. A one-way ANOVA was conducted to examine differences between both groups across all bin intervals. A Benjamini-Hochberg correction was used to reduce type one errors across 18 comparisons. The adjusted *p*-value was set to 0.00284. Four intervals containing negative sentiments reflected a significant difference between both groups, as seen in Figure 4 and Table 5 ([-0.888, -0.777], [-0.555, -0.444], [-0.444, -0.333], [-0.111, 0.000]). While most of the positive sentiment bins did not return a significant *p*-value, the mean values for the Non-PTSD group become increasingly larger than the PTSD group.

**Table 5:** Average binned sentiments per group (bins = 18).

**Bin intervals**			**Non-PTSD**	**PTSD**	***P*-value (Adjusted B-H)**
**Start**	**End**		**Mean ±std**	**Mean ±std**	
-1.000	-0.888		0.266 ± 0.587	0.471 ± 0.828	0.18
-0.888	-0.777		0.734 ± 0.895	1.057 ± 1.178	0.145
-0.777	-0.666		0.814 ± 0.974	1.563 ± 1.499	<0.0001^**^
-0.666	-0.555		1.473 ± 1.435	1.897 ± 1.583	0.234
-0.555	-0.444		1.681 ± 1.146	2.667 ± 2.164	<0.0001^**^
-0.444	-0.333		2.633 ± 2.233	4.425 ± 2.970	<0.0001^**^
-0.333	-0.222		4.255 ± 2.324	5.000 ± 2.512	0.173
-0.222	-0.111		1.362 ± 1.494	1.632 ± 1.399	0.641
-0.111	0.000		32.287 ± 15.669	43.966 ± 19.236	<0.0001^**^
0.000	0.111		1.213 ± 1.125	1.827 ± 1.341	0.003^*^
0.111	0.222		1.803 ± 1.417	2.000 ± 1.742	0.693
0.222	0.333		7.351 ± 4.176	8.023 ± 3.971	0.693
0.333	0.444		8.638 ± 3.986	10.506 ± 4.503	0.008^*^
0.444	0.555		4.218 ± 2.522	4.483 ± 2.832	0.693
0.555	0.666		4.968 ± 3.103	5.667 ± 3.194	0.474
0.666	0.777		4.160 ± 2.477	3.920 ± 2.161	0.693
0.777	0.888		4.261 ± 2.808	3.805 ± 3.013	0.693
0.888	1.000		3.441 ± 3.696	2.529 ± 3.066	0.317

*This shows the average number of sentiments for each bin per group. We conducted a one-way ANOVA to determine statistical differences between all 18 bins. and used a Benjamini–Hochberg correction to reduce type one errors. The corrected statistical significance was set to p <0.00284. Statistical significance denoted by*

* *p <0.00284*,

** *p <0.0001. The bolded row represents the overall high watermark results from our entisre analysis. It simply reflects the highest accuracy across all processes*.

## 5. Discussion

In this study, we sought to distinguish between individuals suffering from PTSD-like symptoms by analyzing sentiment during semi-structured interviews. These interviews were part of a multi-modal dataset, which was used in several data science competitions known as the AVEC challenge. We used a superlearner (SL), which combined different sentiment analyzers, features, and models to best differentiate between PTSD and non-PTSD individuals. We implemented different partitioning methods to determine whether our models could generalize well to unseen data. Our feature engineering method involved binning sentiment scores for each utterance, for each individual, and then concatenating them for a given subject. The SL selected the RF model, using the VADER analyzer, and a bin size of 23, achieving an accuracy of 80.4 % (AUC = 0.80, F1 = 0.79) on the original train-test-split folds given by the AVEC-19 organizers. The partitioning method involving a training set of only 2017 AVEC participants and a test set of 2019 participants also garnered strong results. The RF model with both VADER and Flair achieved accuracies of 80.2 %, and an AUC of 0.82 and 0.81. In the five-fold-CV partition, the best performing model was also the RF with VADER, but with a bin size of 18 (75.6 % accuracy, ± 4.5% STD).

In the five-fold partitioning type, our SL selected the optimal bin size of 18 as part of the final RF model. We examined the distribution of sentiments from the VADER analyzer for both groups in this bin size. We chose to analyze the bin size for the five-fold-CV partitioning, because it served as our most agnostic validation method. Figure 4 revealed that individuals suffering from PTSD had more utterances in the negative sentiment bins compared to the non-PTSD group. However, this trend was slowly reversed as we moved toward the more positive sentiment bins, where non-PTSD individuals had more extremely positive sentiment scores. Our ANOVA test revealed significant differences in many of the negative sentiment bins, but only one in the positive sentiment bins. These results can be seen in Table 5. To summarize, individuals with PTSD seemed to use negative words more often, and they used neutral words more often as well. The only words that controls used more often, were extremely positive.

The results of our binned sentiment analysis are both contradictory and in concordance with several articles regarding emotional arousal in PTSD. On one hand, trauma can induce intense emotions such as anger, fear, sadness, and shame, which can then be reflected through language. On the other hand, individuals faced with intrusive recollections of trauma may avoid emotional eruptions, which could lead to numbness, or they may use substances to circumvent unwanted emotions. Emotional numbing is a biological process where emotions are detached from thoughts, behaviors, and memories (62, 63). Sometimes, a trauma victim may alternate between these two responses like an oscillating rhythm between an overwhelming emotional surcharge and arid states of no feeling at all (64, 65). Based on our results, it seems as though some individuals with PTSD used negative sentiments and neutral sentiments more often than non-PTSD individuals. It may be that both emotional numbing and arousal phases were present and ubiquitous in our sample size. Besides, there is large heterogeneity in behavioral responses to trauma, which may lead to high variance in detecting PTSD through language. Several studies examining the content of traumatic narratives have shown that individuals with PTSD use more emotional words, pronouns, and adjectives (66–68). Pennebaker et al. (66) posited that individuals suffering from suicidal ideation tend to use more emotional language and singular pronouns. The authors suggested that the increase in singular pronouns were reflective of a weakness in communicating with others (66). Another study examining online personal journals surrounding the 9/11 terrorist attacks showed that individuals directly affected by the attacks used stronger negative words, more first-person plural words, and less first-person singular words (69). More recently, a longitudinal study examining 124 9/11 responders sought to predict symptom severity using an interview of their oral history. Cross-sectionally, they found greater negative language and first-person singular usage associated with symptom severity. Longitudinally, they found that anxious language was correlated with higher PCL scores, whereas first-person plural usage and longer words predicted improvement over time (70). The novel finding in this study illustrates that language can be used to predict present and future symptom severity of PTSD. A future goal of ours is to predict symptom severity of individuals with PTSD 6 or 12 months from date of interviews. Regardless, We examined the use of pronouns in our corpus, and found that individuals without PTSD used pronouns more on average. However, individuals with PTSD used a higher proportion of pronouns compared to the rest of the tagged words in their corpus (15 vs. 14%). This is counter to the literature, however we posit that the presence of a virtual avatar may have affected the use of pronouns from individuals with PTSD.

The reason why individuals with PTSD tend to use more negative language is subject to debate. Some theorize that confronting and speaking about unpleasant emotions may help individuals cope with their trauma (71). By reappraising their traumatic experiences, speaking with negative emotions has been shown to mediate autonomic processes that can foster and improve mental wellness (71, 72). This has been studied in exposure therapy (ET), where individuals are repeatedly exposed to anxiety provoking stimuli until their fear response is diminished. During ET, repeated used of emotional language can allow survivors to express their emotions without experiencing the repetitive physiological sensations that come with those emotions over time (73). One study examining emotional narratives of child abuse victims showed an association between strong negative emotional words and the depth of experiencing (meaning exploration), and emotional processing. The authors suggested that the use of strong emotional words is reflective of deeper self-exploration and emotional processing, which can serve as catalysts to construct more positive meaning. Though we do not know the extent, nor the type of therapy some are receiving in our sample, the use of more negative language may stem from a therapeutic mechanism by which emotional language may desensitize physiological symptoms of PTSD (72, 73). Though it is difficult to explain why more negative words were used in the PTSD group, it is possible that some have been taught to use more emotional tones to express trauma-related feelings, which is an important step for recovery.

Determining the type of emotional dysregulation early in the diagnostic procedure may benefit in tailoring specific treatments to help regulate affective responses. As mentioned, there is large heterogeneity in behavioral responses following traumatic events. Some survivors display a high degree of emotional resilience, while others go on to develop PTSD. Therefore, it is important for a clinician to reliably determine who may need therapeutic intervention, and allocate resources to those who need it most (74). Natural language and sentiment analysis can serve as markers to facilitate initial assessments, and can possibly be used to tailor future treatments such as mindfulness, pharmacotherapy, cognitive structuring, and trauma-specific desensitization techniques such as exposure therapy or eye movement desensitization reprocessing (EMDR) (62, 75, 76). Additionally, text-based analysis may be used to predict symptom severity several months from initial assessment (74). Linguistic markers such as emotional language can be a non-invasive, cost-effective, rapid modality that will complement the current trend into telepsychiatry. Here, we have shown that linguistic markers such as sentiment analysis can be used to identify individuals suffering from PTSD (77).

The present study illustrates how ML techniques can be used to produce models that can identify individuals suffering from PTSD using teleconferencing interviews. Implementation and practicality of such learned models have become more accurate over the last 10 years. One meta-analysis examining classification studies showed that 41 of 49 articles achieved an accuracy of at least 83.7% when it came to predicting which individuals suffer from PTSD (15). Granted, these studies used different modalities such as functional neuroimaging, facial/motion tracking, and speech signals. The authors also note that learned models outperform many standard methods due to their sensitivity for hidden interactions and latent variables between predictors. They can also account for non-linear patterns in a dataset (15). Beyond that, computational power has allowed for models to handle large, heterogeneous sources such as audio/video recordings, biological samples, and neuroimaging. With enough data, these models will increase their predictive power, which may help identify certain mental conditions or even suggest certain therapies.

This study has several limitations. Firstly, the sample size is only 275 participants, with only 23 % having PTSD. In our training sets, we used up-sampling to handle imbalanced classes. Ideally, a much larger dataset with more PTSD individuals could help produce a model with more robust predictions. Secondly, though the PCL-C is a reliable self-report measure, it is not considered the gold standard for diagnosing PTSD. The CAPS-5 is a clinically structured interview that is globally used to detect presence of PTSD. This would have been the preferred outcome measure for this study. Thirdly, we did not examine the presence of an artificial avatar (Ellie) conducting the interview. Though speculating, we believe that responding to an avatar may change the emotional dynamic by which an individual uses to communicate. An interesting study would look at the differences in emotional language between a human therapist and an avatar. Fourth, while we showed our model is generalizable by examining multiple partitioning methods within the AVEC-19 dataset, we do not have an external dataset (from another location) to test our model on. It is difficult to say whether our model is generalizable without testing it on a different set of participants. Lastly, a deeper analysis on part-of-speech tagging (POS) should have be conducted in future studies. We found several studies citing an association with POS categories and severity of PTSD symptoms. Part-of-speech tagging is a type of NLP which identifies the category of spoken words in a text. By categories, we refer to nouns, pronouns, adverbs, and so on. A future study should consider POS tagging features to predict PTSD instead of only emotional language.

Overall, our study showed that extracting sentiment from natural language is sufficient to detect individuals suffering from PTSD. Our analysis can be used solely, or be extended to include additional modes of data such as speech signals or motion tracking. We intend on doing this in future studies. As mentioned previously, quantifying emotional expression, valence, and arousal for the purpose of diagnosis or symptom monitoring has had several issues: (1) Self-report measures rely on retrospective client or clinical insight, and do not capture emotional changes across several sessions. (2) Clinician ratings can be more objective (kappa value <0.7), but require time intensive input and coding (78–80). However, recent advances in ML and telepsychiatry may provide an opportunity for sentiment analysis to be implemented into online assessments and therapeutic sessions. Using ML models to detect emotion in assessments can assist in determining a diagnosis, it can be used to establish a therapeutic alliance, and it can reflect behavioral changes over time (78, 81–83). Linguistic tools such as this may be used in conjunction with self-reports and interviews to better detect PTSD. With the current global pandemic, PTSD rates may continue to rise, thus accessible and accurate assessments of PTSD may be needed.

## Data Availability Statement

The data analyzed in this study is subject to the following licenses/restrictions: The Distress Analysis Interview Corpus is privately available to only academics and non-profit researchers. Please visit https://dcapswoz.ict.usc.edu/ to apply. Requests to access these datasets should be directed to Jill Boberg, boberg@ict.usc.edu. Our code for this project can be found at https://gitlab.com/jsawalha/avec-19_sentiment_analysis.

## Ethics Statement

The studies involving human participants were reviewed and approved by University of South California—Review Board University of Alberta—Health Research Ethics Board. The patients/participants provided their written informed consent to participate in this study.

## Author Contributions

JS and MY: investigation, data analysis, writing—original manuscript, and editing. MB and ZS: reviewing and editing. AG and RG: principal investigators, project supervisors, final review, and editing. All authors contributed to the article and approved the submitted version.

## Conflict of Interest

The authors declare that the research was conducted in the absence of any commercial or financial relationships that could be construed as a potential conflict of interest.

## Publisher's Note

All claims expressed in this article are solely those of the authors and do not necessarily represent those of their affiliated organizations, or those of the publisher, the editors and the reviewers. Any product that may be evaluated in this article, or claim that may be made by its manufacturer, is not guaranteed or endorsed by the publisher.
